# Anti-infective treatment in HIV-infected patients during perioperative period

**DOI:** 10.1186/1742-6405-9-36

**Published:** 2012-11-27

**Authors:** Baochi Liu, Lei Zhang, Ruizhang Guo, Jinsong Su, Lei Li, Yanhui Si

**Affiliations:** 1 Department of Surgery, Shanghai Public Health Clinical Center Affiliated to Fudan University, 2901 Caolang Road, Shanghai, Jinshan District, 201508, China; 2 Department of Stomatology, Shanghai Public Health Clinical Center Affiliated to Fudan University, 2901 Caolang Road, Shanghai, Jinshan District, 201508, China; 3 Department of Emergency, The First Affiliated Hospital of Zhengzhou University, No.1 Jianshe Road, Zhengzhou, 450052, China

**Keywords:** HIV/AIDS, Surgical site infection, Sepsis, Anti-infective treatment

## Abstract

**Objective:**

To investigate anti-infective treatments in HIV-infected surgical patients during the perioperative period.

**Methods:**

A retrospective study of sepsis and surgical site infections (SSIs) was conducted in 266 HIV-infected patients. The patients were divided into 3 groups based on CD4+ T cells counts in the preoperative period: group A (0–199 cell/ul), group B (200–349 cell/ul) and group C ([greater than or equal to] 350 cell/ul). When the CD4 count was below 350 cells/uL, anti-retrovirus therapy was started. For patients whose preoperative CD4 counts were [less than or equal to] 200 cells/uL, preoperative antibiotic medication was started.

**Results:**

Patients in group A were more likely to get sepsis than patients in the other two groups (p0.01). Among 82 patients with clean wounds, only one patient got SSIs. All patients with dirty wounds had acquired SSIs after surgery. There were only 6 patients dead at 30 days after surgery, a death rate of 2.3%. Sepsis appeared in 110 patients (41%).

**Conclusions:**

Complete evaluation of surgical risk and suitable perioperative anti-infective treatment may lead to better outcome for HIV-infected surgical patients.

## Introduction

The patient who undergoes surgical treatment may experience postoperative infection. According to wound class and immune status, the postoperative infection rates are different. Based on the National Nosocomial Infections Surveillance (NNIS) system, surgical site infections (SSIs) are the third most frequently reported nosocomial infection, and the most common on surgical wards [1]. The Centers for Disease Control and Prevention (CDC) estimates that 22% of all health-care-associated infections are SSIs, with an increasing percentage over the last decade [1,2]. SSIs increase morbidity as well as mortality, double the length of hospital stay [3-6] and increase the cost of surgery two- to five-fold [7].

The progressive failure of the immune system caused by HIV can increase the possibility of developing postoperative infection. In recent years, the number of HIV-infected patients is progressively increasing. The introduction of antiretroviral therapy (ART) has significantly improved the life expectancy of patients infected with HIV and those diagnosed with AIDS [8-13]. The demand for surgical treatment in HIV-infected patients is increasing and so is the frequency of resultant surgical disease [14,15].

There are few reported data on the efficacy of anti-infective treatment in HIV-infected patients during the perioperative period. Since our institution is a designated tertiary care university hospital for treatment of HIV-infected patients we had the opportunity to investigate the efficacy of anti-infective treatments in HIV-infected patients during the perioperative period.

## Patients and methods

### Definitions

We defined the wound class at the surgical site of infection (SSI) using the definitions provided by the Centers for Disease Control and Prevention (CDC). An instance of surgical site infection was defined as an infection occurring within 30 days of the operative procedure, when the patient had one or more of the following: (1) organisms isolated from an aseptically obtained culture of fluid or tissue from the surgical incision; (2) purulent drainage from the surgical incision; (3) at least one of the following: pain or tenderness, localized swelling, redness, or heat. SSIs were classified as being incisional, deep incisional or organ/space. Surgical procedures were classified as clean, contaminated or dirty. Sepsis was defined as the infection in association with systemic inflammatory response syndrome.

### Data collection

Clinical data of HIV-infected patients undergoing surgery from January 2009 to December 2011 were retrieved using our computerized patient record system. Inclusion criteria: HIV-infected patients were identified and diagnosed by local Centers for the Disease Prevention and Control in different locations in China. All selected patients had records that contained thorough disease histories, including physical examinations, preoperative and postoperative routine examinations, and immune function tests.

### Patient group and study methods

We stratified and compared the incidence of SSIs and sepsis according to both wound class and preoperative CD4 counts with breakpoint values of 200 and 350 cell/μL. Demographic and clinical information was entered into a database that included: type of surgical procedure, age, peripheral blood cell counts, plasma albumin, CD4 counts, and CD4/CD8 ratios.

### Statistical analysis

Date were analyzed using SPSS 16.0 statistical software (SPSS Inc., Chicago, IL). Results of all continuous data are presented here as mean ± standard deviation. Continuous variables were compared by independent t-test. Univariate analysis of the categorical outcome was carried out using Chi-squared tests. P<0.05 indicates statistical significance.

## Results

Two hundred and sixty-six HIV-infected patients were included in this study. Their mean (±SD) age was 42.3 (±13.1) years, and 237 (89.1%) were male. There were six deaths in the study population, five of whom had organ/space SSI and developed sepsis. The SSI cumulative incidence rate was 46.6% (124 of 266). The incidence of incisional SSI was 37.6%, deep incisional SSI was 5.3%, and organ/space SSI was 3.8%. Baseline characteristics of patients shown in (Table 1).

**Table 1:** Baseline characteristics of patients

**Variable**	**No.**	**Percent of total study population**
Age; median(IQR), yr	42.3 (10–74)	
Mortality(death during hospitalization)	6	2.3
Male sex	237	89.1
Comorbidities		
Heart disease	0	
Diabetes	3	1.1
Cancer	47	17.7

Univariate analysis revealed that the following variables were associated with the occurrence of sepsis: preoperative WBC, hemoglobin, CD4, CD8 and CD4/CD8 (Table 2).

**Table 2:** Comparisons of age and clinical date between sepsis group and non-sepsis group

**Parameters**	**Sepsis group**	**Non-sepsis group**	**t**	**P**
Age(y)	41.8 ± 12.7	42.7 ± 13.8	−0.529	0.598
Viral load	105027.01 ± 2.58*10^5^	46203.79 ± 1.02*10^5^	1.071	0.26
WBC(x10^9^/L)*	6.15 ± 3.317	5.23 ± 1.79	2.625	0.010
Hemoglobin (g/L)*	113.7 ± 24.9	130.1 ± 23.2	−5.349	0.000
Platelet (x10^9^/L )	213.4 ± 114.5	190.51 ± 75.19	1.821	0.070
CD4 (cell/ul)*	172.6 ± 163.4	324.01 ± 242.64	−5.349	0.000
CD8 (cell/ul)*	666.9 ± 485.4	798.43 ± 424.348	−2.132	0.034
CD4/CD8*	0.30 ± 0.25	0.4532 ± 0.3129	−3.836	0.000

* Statistical significance.

At multivariate analysis, only preoperative WBC, hemoglobin and CD4 remained significantly (P<0.05) associated with sepsis, (Table 3).

**Table 3:** Independent risk factors for perioperative sepsis estimated from multiple regression model

**Risk factor**	**OR**	**95%CI**	**P value**
Preoperative CD4*	6.069	1.001-1.010	0.014
Preoperative CD8	0.469	0.998-1.001	0.494
Preoperative CD4/CD8	0.2 → 1	0.027-8.425	0.616
Preoperative Hemoglobin *	4.302	1.001-1.036	0.038
Preoperative Albumin	0.12	0.962-1.028	0.729
Preoperative WBC*	5.814	0.719-0.996	0.016

Comparisons of the incidence of sepsis according to preoperative CD4 counts shown in Table 4.

**Table 4:** Incidence of postoperative sepsis according to preoperative cd4 counts

***Preoperative CD4***	***Sepsis***	***Non-sepsis***	***χ***^***2***^	***p***
<200 cell/μl	68 (66.7%)	34 (33.3%)	41.404	<0.01*
200-349 cell/μl	18 (28.6%)	45 (71.4%)		
≥350 cell/μl	13 (20.6%)	50 (79.4%)		

* Statistical significance.

Patients with lower preoperative CD4 counts undergoing surgery were more likely to develop sepsis.Surgical procedures were classified as clean (N = 82; 30.8%), contaminated (N = 171; 64.3%) and dirty (N = 13; 4.9%). The incidence of SSI differed significantly depending on wound class, and increased from 1.2% in patients with clean wounds to 100% in patients with dirty wound (Table 5).

**Table 5:** Incidence of SSIs according to wound class

**Wound class**	**Non-SSIs**	**SSIs**	***χ***^***2***^	**p**
clean	81 (98.8%)	1 (1.2%)	104.349	<0.001*
contaminated	61 (35.7%)	110 (64.3%)		
dirty	0	13 (100%)		

* Statistical significance.

Our data show that SSIs were frequent and differed widely by wound class. Patients with SSIs were more likely to develop postoperative sepsis than non-SSIs patients (Table 6).

**Table 6:** Incidence of postoperative sepsis according to SSIs

	**Sepsis**	**Non-sepsis**	***χ***^***2***^	***p***
SSIs	63(50.8%)	61(49.2%)	8.559	0.003*
Non-SSIs	47(33.1%)	95(66.9%)		

* Statistical significance.

## Discussion

### Anti-infective treatment in the perioperative period

HIV mainly invades and destroys CD4 T lymphocytes, causing CD4 counts to gradually decrease. In consequence of their significantly impaired immune function when the^,^CD4 count is below 350 cell/μL, HIV-infected patients are likely to get opportunistic infections and cancers and their mortality is then usually high [16]. At this stage **ART** is used, and usually includes two kinds of nucleosides and one kind of protease inhibitor. Patients using **ART** who undergo surgery need to continue **ART** use during the perioperative period [17]. Even if patients are undergoing gastrointestinal surgery and need to fast, they can take antiviral drugs. When a patient's^,^ CD4 count is below 200 cell/μL, there is an additional high risk of acquiring fungal infections such as *Pneumocystis carinii* pneumonia which bring a marked increase in mortality [18,19]. Therefore SMZ and fluconazole are conventionally added to **ART** during the perioperative period to prevent *P. carinii* pneumonia and other fungal infections. If tuberculosis, cryptococcosis, candidiasis or similar infections have been found before surgery, anti-TB or antifungal treatments are clearly required to control the disease. In summary, HIV patients undergoing emergency surgery need prophylactic anti-infective drugs based on doctor/clinical experience, and effective anti-infective treatments are to be applied according to the results of resection of lesions and blood culture [20,21].

### Prophylactic antibiotics

To reduce the incidence of surgical site infection, prophylactic antibiotics are generally used during the perioperative period. Because of the weakened immune function, HIV-infected patients are even more likely to need prophylactic antibiotics [22]. However, there are no reports specifically about how to use antibiotic in surgical patients who are HIV infected. We found that preoperative CD4 lymphocytes, WBC and hemoglobin are independent risk factors for sepsis. CD4 counts cannot be implied from white blood cell counts. Decline in CD4 T lymphocyte counts is often accompanied by decline in hemoglobin. Our principle in using prophylactic antibiotics was to use those antibiotics which can cover the most common infections according to surgical incision site and type of surgery. We took into account the likelihood of Gram-negative bacilli (enteric bacteria), gram-positive cocci (*Staphylococcus aureus*) and anaerobic bacteria, and chose between two kinds of antibiotic combinations. We usually selected first-generation cephalosporins on clean wound surgery. For nine patients who were undergoing excision of thyroid tumor or breast tumor, we did not use any antibiotics and no SSI arose. In all, 82 patients underwent clean wound surgery and 81 had healing wounds. In future we may try not using any antibiotic for classIminor incisions. For giant splenectomy with cirrhosis and for internal fixation of femoral fractures, we still need prophylactic antibiotics, and they should be used longer than for normal surgery. We generally use antibiotics until wound have healed. The first-generation cephalosporins have a strong bactericidal activity for Gram-positive *S. aureus*, but for Gram-positive intestinal bacteria its bactericidal activity is less than second- and third-generation cephalosporins (ceftriaxone, etc.). Second-generation cephalosporins have advantages in the prevention of wound infection for class-IIincisions and gastrointestinal tract surgery, but its bactericidal activity is less than third-generation cephalosporins in the prevention of intra-abdominal infection. Prophylactic antibiotics should also cover common anaerobic bacteria for lower gastrointestinal surgery where there is significant pollution. Antibiotics such as piperacillin, cefoxitin, cefotetan, etc., can cover gram-negative enteric bacilli as well as anaerobic bacteria. Metronidazole and clindamycin should be included when other antibiotics do not have activity against anaerobic bacteria.

### Antibiotics for therapy

Therapeutic drugs should be used for intra or postoperative infections. According to our statistical analysis of the clinical data, the lower the preoperative CD4 counts, the higher the incidence of sepsis. Our data also show that SSIs were frequent and differed widely by wound class. The incidence of SSIs is high for Class II incisions because our study included many anal warts excision patients and these usually develop SSIs after surgery. Clearly, we should maintain clean wounds for superficial surgical site infections to reduce sepsis; our data show that the incidence of sepsis in the SSIs group was significantly higher than in the non-SSIs group. Therefore effective treatment should also be used for surgical site infection. Effective anti-infection treatment involves a multi-disciplinary knowledge for HIV-infected patients who may have tuberculosis, fungal infections and surgical site infections.

### Surgical risk and prognosis

HIV-infected patients have a higher incidence of surgical complication and mortality than normal patients. Jeremiah L et al. [23] reported that the incidence of postoperative infectious complications was 55% and the 30-day mortality rate after surgery was 30% for HIV-infected patients. Our institution is a designated tertiary care university hospital for treatment of HIV-infected patients, so we have accumulated much perioperative anti-infection treatment experience. The mortality of HIV-infected patients undergoing abdominal surgery in our hospital was 7.7% which is significantly lower than previously reported data. Surgeons should pay attention to occupational exposure and aseptic technique in order to reduce SSIs and surgical trauma.

### Limitations of the study

One of the limitations of the study was possible information biases due to the retrospective nature of the study design. Another was that the study did not control for possible confounders other than those investigated.

In conclusion, in order to reduce the incidence of infection complications and mortality, surgeons should combine multidisciplinary knowledge and carry out reasonable anti-infection treatments.

### Ethical approval

The study was approved by the Ethical Committee of Shanghai Public Health Clinical Center, Fudan University, Shanghai, China. (International index IORG0006364).

## Competing interests

The authors have no financial or other conflicts of interest regarding this article.

## Authors’ contributors

BL, LZ and GZ conceived of the study, participated in its design and draft the manuscript. LL and JS participated in data collection. All authors read and approved the final manuscript.
